# 

**Published:** 1888-09

**Authors:** 


					L  SEPTEMBER, 1888.
v
No. 21.
?E /""smmy'
\ ' N' DEXE
BRISTOL
MEDICO-CHIRURGICAL
JOURNAL
a <&uarterls Journal of tbe /nbeOtcal Sciences for tbe "Wllest
of Bnglanfc aitfc Soutb Tldales
PUBLISHED UNDER THE AUSPICES OF
THE BRISTOL MEDICO-CHIRURGICAL SOCIETT.
EDITOR :
J. GREIG SMITH, M.A., F.R.S.E.
ASSISTANT-EDITOR :
L. M. GRIFFITHS.
"Scire est nescire, nisi i& me
Scire alius sciret."
BRISTOL: J. W. ARROWSMITH ;
London: J. & A. Churchill, 11 New Burlington Street.
PW/>? f)na RMl.lina and Sixvence.

				

